# Pattern Recognition and Signaling Mechanisms of RIG-I and MDA5

**DOI:** 10.3389/fimmu.2014.00342

**Published:** 2014-07-23

**Authors:** Stephanie Reikine, Jennifer B. Nguyen, Yorgo Modis

**Affiliations:** ^1^Department of Molecular Biophysics and Biochemistry, Yale University, New Haven, CT, USA

**Keywords:** pathogen-associated molecular pattern, nucleic-acid sensor, RecA-like DEAD-box (DExD/H-box) RNA helicase, caspase recruitment domain, signal transduction, signalosome, prion-like switch, amyloid-like aggregation

## Abstract

Most organisms rely on innate immune receptors to recognize conserved molecular structures from invading microbes. Two essential innate immune receptors, RIG-I and MDA5, detect viral double-stranded RNA in the cytoplasm. The inflammatory response triggered by these RIG-I-like receptors (RLRs) is one of the first and most important lines of defense against infection. RIG-I recognizes short RNA ligands with 5′-triphosphate caps. MDA5 recognizes long kilobase-scale genomic RNA and replication intermediates. Ligand binding induces conformational changes and oligomerization of RLRs that activate the signaling partner MAVS on the mitochondrial and peroxisomal membranes. This signaling process is under tight regulation, dependent on post-translational modifications of RIG-I and MDA5, and on regulatory proteins including unanchored ubiquitin chains and a third RLR, LGP2. Here, we review recent advances that have shifted the paradigm of RLR signaling away from the conventional linear signaling cascade. In the emerging RLR signaling model, large multimeric signaling platforms generate a highly cooperative, self-propagating, and context-dependent signal, which varies with the subcellular localization of the signaling platform.

## Introduction

Eukaryotic organisms rely on their innate immune system to detect viruses and other microbes. Innate immune receptors detect chemical patterns or structures that are broadly conserved in microbes, including bacterial cell wall components, microbial nucleic acids, and certain highly conserved proteins. These pathogen-associated molecular patterns (PAMPs) are recognized by pattern recognition receptors that fall into several families, including Toll-like receptors (TLRs), NOD like receptors (NLRs), C-type lectin receptors (CLRs), and RIG-I-like receptors (RLRs). At the cell surface and in endocytic compartments, TLRs are the most important family of molecular sentries for the innate immune recognition of a wide range of microbial patterns outside the cytosol (1). CLRs, such as Dectin1, are localized on the cell surface and principally recognize fungal pathogens (2). In the cytosol, NODs and other NLRs recognize cell wall fragments and other bacterial components (3). This review will focus on the RLRs, which are found in the cytosol and recognize viral double-stranded RNA (dsRNA). Innate immune receptors from all families have in common that they nucleate the assembly of large multimeric protein complexes with their signaling adaptors, which include most notably MyD88, MAVS, ASC, and RIP2 (4). These oligomeric assemblies rapidly activate and amplify potent inflammatory antimicrobial responses, principally through the activation of NF-κB, type I interferons, or caspase 1.

**Figure 1:**
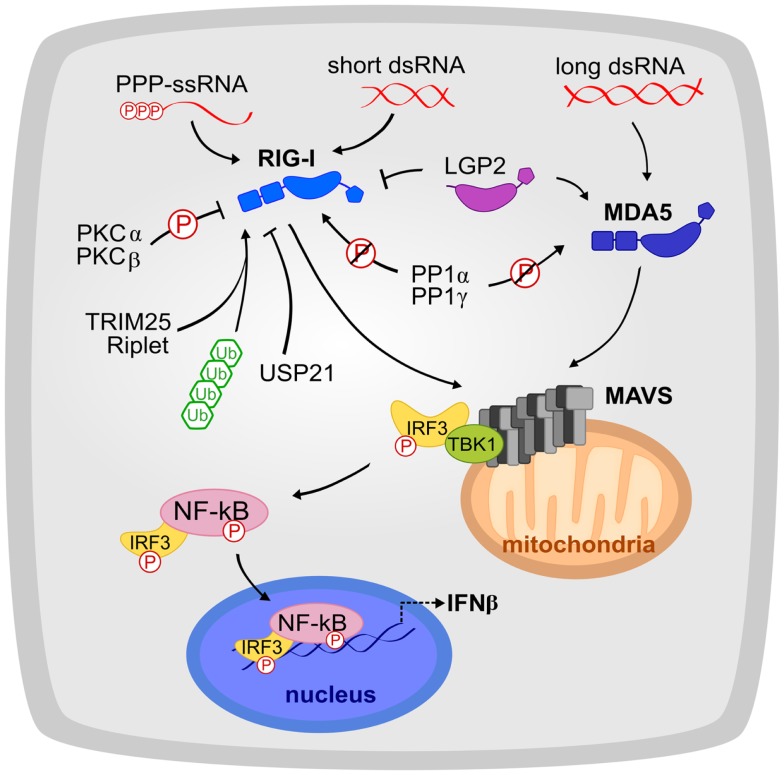
**The RLR signaling pathway is shown**. RIG-I and MDA5 recognize a complementary set of cytosolic viral dsRNA ligands. Their activation is tightly regulated by phosphorylation, ubiquitination, and host proteins such as LGP2. RIG-I and MDA5 signal to MAVS, which initiates the production of interferon signaling. Circled “P” indicates phosphorylation and slashed circled “P” indicates dephosphorylation.

Nucleic acids are the largest, and arguably the most important class of ligands for innate immune receptors. To avoid signaling in response to endogenous nucleic acids, which are ubiquitous in the cytoplasm and nucleus, innate immune sensors must recognize specific patterns in specific subcellular locations. (1) A subfamily of TLRs (TLRs 3, 7, 8, and 9) recognizes microbial DNA and RNA ligands exclusively in endolysosomal compartments (5–9). In the cytosol, two essential immune sensors, RIG-I and MDA5, detect viral dsRNA (10–12). Several different sensors recognize double-stranded DNA (dsDNA) in the cytoplasm, including proteins from the AIM2 family, the DDX family, RNA polymerase III, and cyclic GMP–AMP synthase (13, 14). Ligand binding by each of these sensors induces a conformational change that directs the cooperative assembly of large oligomeric signaling platforms, leading to the recruitment and activation of signaling adaptors (4). The rapidly ensuing inflammatory response culminates in activation of the NF-κB and type I interferon signaling pathways (Figure 1). This response is one of the first and most important lines of defense against infection and is responsible for the activation of the adaptive immune system (1). Innate immune receptors therefore play pivotal roles as master-regulators of inflammation.

**Figure 2:**
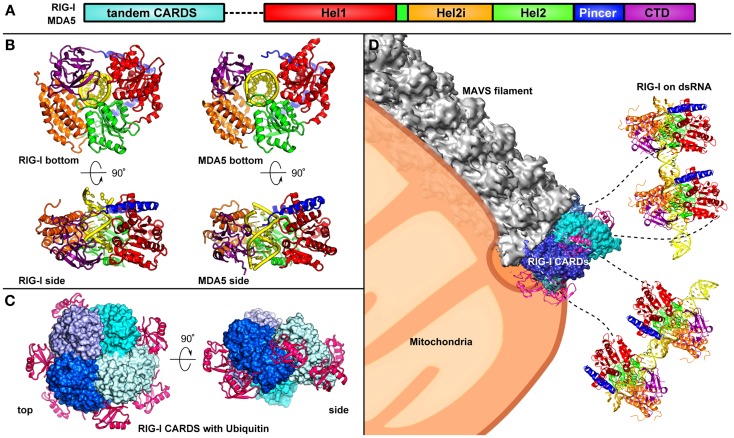
**Assembly of the RLR signalosome is shown**. **(A)** The domain architecture of RIG-I [colored as in Ref. (34)]. **(B)** Two orthogonal views of the RIG-I (left) and MDA5 (right) helicase domains and CTD bound to a dsRNA ligand (17). The CTD of RIG-I caps the 5′ end of the dsRNA ligand, however, in MDA5 the CTD is rotated by 20° relative to Hel2, allowing for MDA5 to polymerize along the dsRNA. **(C)** Two orthogonal views of the RIG-I tandem CARDs, which assemble into a “lock-washer” with three K63-di-ubiquitin molecules are shown (35). **(D)** RIG-I recognizes viral dsRNA in the cytosol and undergoes a conformational change, releasing the CARDs from an auto-repressed state. Four RIG-I molecules come together and their CARDs assemble into an oligomer stabilized by unanchored K63-linked polyubiquitin chains. The RIG-I CARDs serve as a scaffold for MAVS, which forms a filament that is tethered on the mitochondrial or peroxisomal membrane (36).

Many viruses deliver an RNA genome into the cytoplasm or rely on a replication or transcription step that generates viral RNA in the cytoplasm. Infection by these viruses is primarily detected by RIG-I and MDA5, also referred to as the RLRs. RIG-I and MDA5 sense complementary sets of viral RNA ligands (10–12, 15). RIG-I recognizes 5′-phosphorylated blunt ends of viral genomic dsRNA, whereas MDA5 binds internally to long dsRNA with no end specificity (10–12). RIG-I and MDA5 both have tandem N-terminal caspase recruitment domains (CARDs) with death domain folds, a DExD/H-box helicase (consisting of two RecA-like helicase domains, Hel1 and Hel2, and an insert domain, Hel2i), and a C-terminal domain (CTD) (Figure 2A). In the absence of dsRNA, RIG-I has a closed inactive conformation (16). RNA binding through the helicase and CTD domains (17, 18) releases the CARDs, which then recruit and activate the signaling adaptor MAVS (IPS-1) (19). In contrast, MDA5 does not sequester its CARDs (20) and cooperatively assembles into ATP-sensitive filaments on dsRNA (20–22). Moreover, the MDA5 CTD is required for cooperative filament assembly but not for RNA binding (20, 23, 24). The MDA5 CARDs have been proposed to nucleate the assembly of MAVS into its active polymeric form (20, 25) in a process that can be promoted by K63-linked polyubiquitin chains (26). The self-propagating amyloid-like properties of MAVS polymers amplify signaling (25). RLR signaling is regulated by numerous host and viral factors through various mechanisms, including ubiquitin-dependent proteolytic degradation and cleavage of MAVS by virally encoded proteases (27–29). A third RLR, LGP2, lacks CARDs and exerts co-stimulatory and inhibitory functions on MDA5 and RIG-I, respectively (30–33).

Recent biochemical, biophysical, and cellular studies have greatly advanced our understanding at the molecular level of the mechanisms of pattern recognition and signaling by RIG-I and MDA5. Here, we review these studies and their implications on the current models of microbe-induced inflammation, auto-inflammation, and inflammation-induced cancer.

## Recognition of dsRNA in the Cytosol by RIG-I and MDA5

### The molecular determinants of ligand recognition by RLRs

RIG-I preferentially binds to short (<300 bp) dsRNAs that have blunt ends and a 5′ triphosphate (5′-ppp) moiety, facilitating discrimination between host and viral dsRNA (10–12). Crystal structures of RIG-I bound to a 12-bp dsRNA ligand and of unliganded RIG-I have provided detailed insights into the mechanism of activation of this receptor. In the absence of dsRNA ligand, RIG-I is in an auto-repressed state: the domains in the helicase domain are in an open conformation and the tandem CARDs form contacts with the Hel2i domain. This conformation sterically prevents the CARDs from binding to polyubiquitin or to CARDs from other binding partners, thereby preventing signaling to MAVS (16).

Upon the presentation of a viral dsRNA, RIG-I undergoes significant conformational rearrangement. The CTD binds tightly to the 5′-ppp and the helicase domains wrap around dsRNA, adopting a more compact configuration (16–18) (Figure 2B). RIG-I recognizes RNA primarily through non-specific interactions with the phosphate sugar backbone, predominantly by the Hel2i domain. This conformational change allows ATP to bind RIG-I, a necessary step for the activation of RIG-I (16–18). Although the CARDs were absent from the RNA-bound RIG-I crystal structures, biochemical studies and small angle X-ray scattering data indicate that the tandem CARDs are released from the Hel2i domain in the active form of RIG-I (17, 18).

In contrast to RIG-I, MDA5 preferentially binds internally to long dsRNA (>1,000 bp) with no end specificity (10–12) and cooperatively assembles into a filament on the dsRNA (20, 21). Unlike RIG-I, the CARDs of MDA5 are not sequestered in the absence of ligand (20). The forced proximity of the CARDs upon MDA5 filament formation induces oligomerization of MDA5 CARDs, forming a scaffold for binding and oligomerization of MAVS CARD (see Activation of MAVS and Downstream Signaling). Notably, the atomic structures of the MDA5 CARDs have not yet been determined.

A crystal structure of the MDA5 helicase domains and CTD bound to dsRNA revealed how MDA5, despite having a similar domain architecture as RIG-I, recognizes dsRNA in a different manner (Figure 2B). The helicase domains of MDA5 wrap around dsRNA similarly to the helicase domains of RIG-I (34, 37). However, consistent with the observation that MDA5 is not preferentially activated by 5′-ppp dsRNA (10–12), the MDA5 CTD is rotated by 20°, bringing it closer to the dsRNA, as compared to the RIG-I structure. The CTD also forms contact with Hel1 in MDA5, such that MDA5 forms a closed ring around the dsRNA (37). This orientation of the CTD promotes cooperative filament formation along dsRNA, initiated from internal sites in the dsRNA rather than from one of the ends (20, 21, 34).

The RLRs are part of the DExD/H-box helicase family based on their domain architecture (33), but they do not appear to have dsRNA helicase activity. Instead, ATP binding and hydrolysis have been implicated in filament formation. ATP binding strengthens the interaction between MDA5 and the dsRNA (34). ATP hydrolysis, however, causes MDA5 to dissociate from the dsRNA (20, 38). At the ends of the MDA5-RNA filaments, ATP hydrolysis causes depolymerization, providing a mechanism for shutting down the signal and for recycling of MDA5. MDA5 filament assembly and disassembly dynamics provide the specificity for long dsRNA (20, 38). RIG-I was also shown recently to form ATP-dependent filaments, although the RIG-I filaments are shorter and less stable than MDA5 filaments (34, 39).

LGP2, the third RLR, has similar helicase and CTD domains as RIG-I and MDA5, but it lacks the tandem CARDs (33). LGP2 recognizes the termini of dsRNA through similar types of protein-RNA contacts as RIG-I and MDA5 (23, 33, 40, 41). ATP hydrolysis enhances RNA recognition by LGP2 (42). Because it does not have CARDs, LGP2 does not recruit MAVS or induce MAVS signaling. LGP2 affects signaling in response to viral stimuli, however, by modulating the RIG-I and MDA5 signals (see Regulation of RLR Signaling) (30–33).

### Role of unanchored lysine 63-linked ubiquitin chains in RLR activation

The oligomerization of the RNA sensors RIG-I and MDA5 that activates the antiviral innate immune response depends on unanchored lysine 63-linked polyubiquitin chains (19, 26). In 2010, Chen and colleagues reconstituted the RIG-I pathway *in vitro* and demonstrated that unanchored K63-linked polyubiquitin chains are required for a full signaling response as measured by IRF3 dimerization (19). Polyubiquitin chains containing as few as four ubiquitin molecules bind non-covalently to the RIG-I CARDs and can be covalently attached to RIG-I by the E3 ligase TRIM25 (19, 43). Furthermore, RIG-I interacted with K63-linked polyubiquitin chains from HEK293T cells in co-immunoprecipitation experiments (19). Similar studies generalized these findings to MDA5 and showed that K63-ubiquitin chains promoted oligomerization of the MDA5 CARDs (26).

A recent crystal structure of the tandem CARDs of RIG-I bound to K63-diubiquitin revealed the molecular basis of the CARD-ubiquitin interaction (Figure 2C) (35). K63-ubiquitin chains promote the assembly of RIG-I CARDs into a tetrameric “lock-washer” structure by stabilizing intermolecular CARD–CARD interactions. This RIG-I tetramer recruits and activates MAVS (see next section) (35). Monoubiquitin is not sufficient to promote RIG-I signaling because a single ubiquitin domain does not make enough contacts to significantly stabilize RIG-I oligomerization through CARD–CARD interactions (19, 35).

Although ubiquitin chains promote RIG-I tetramerization, RIG-I and MDA5 can both assemble into oligomeric filaments and induce MAVS filament formation and signaling in the absence of polyubiquitin chains. Indeed, under certain experimental conditions, namely in the absence of polyubiquitin and as a result of ATP hydrolysis, RIG-I has been observed to form filaments along dsRNA (34, 39, 44). Similarly, MDA5 signaling is thought to be triggered by the formation of MDA5 filaments along dsRNA, which is a ubiquitin-independent process (20, 21). The forced juxtaposition of RLR CARDs upon RLR filament formation is thought to be sufficient to activate MAVS signaling (34). Both RIG-I CARDs and MDA5 CARDs have, however, been shown to bind K63 polyubiquitin chains (26). Hence the question arises of whether K63-linked ubiquitin chains always participate in RLR signaling, or whether they are only required under specific physiological conditions that do not favor RLR filament formation. Because RIG-I has much higher affinity for the 5′-ppp end of viral ligands than it does for the phosphate backbone alone, it has been proposed that RIG-I is more likely to bind to the 5′-ppp end of the dsRNA (34). If sufficient polyubiquitin is available, RIG-I does not form a filament and instead remains at the end of the dsRNA, and the tetrameric CARD lock-washer scaffold is formed (34, 35). K63-linked polyubiquitin chains stabilize the CARDs oligomer through non-covalent interactions. Covalent linkage of the ubiquitin chains to RIG-I by TRIM25 can provide further stabilization of the RIG-I oligomer, thereby increasing interferon signaling capacity (19, 35, 43). If the local concentration of polyubiquitin is insufficient to induce RIG-I CARDs tetramer formation, ATP hydrolysis may enable RIG-I to translocate along dsRNA and assemble into filaments (39), bringing the CARDs together by cooperative stacking of the helicase domains and leading to ubiquitin-independent signal activation. Unlike RIG-I, MDA5 has no known RNA end-preference and MDA5 has a higher propensity to form filaments than RIG-I (26, 34). Hence, the physiological role of unanchored polyubiquitin chains in MDA5 signaling remains less well understood than in RIG-I.

## Activation of MAVS and Downstream Signaling

In the textbook view of RLR signaling, the signal is propagated sequentially from the ligand-bound RLR to MAVS to the cytosolic protein kinases IKK and TBK1, which in turn activate the transcription factors NF-κB and IRF3, respectively (45). Activated NF-κB and IRF3 are translocated into the nucleus, where they induce expression of type I interferons and other inflammatory antimicrobial molecules. The discovery that ligand binding induces RIG-I and MDA5 to assemble into large oligomeric platforms with MAVS on the mitochondrial and peroxisomal membranes has, however, shifted the paradigm for RLR signaling away from the model of a linear signaling cascade. As reviewed in the previous section, both RIG-I and MDA5 form filaments along dsRNA ligands. For RIG-I the forced juxtaposition of its CARDs, along with binding of K63-linked polyubiquitin chains, promotes the formation of a tetrameric lock-washer structure (Figure 2C), which serves as a platform to recruit MAVS (35). Structural and biochemical data suggest that the minimal signaling unit for MDA5 is much larger than for RIG-I and contains at least 11 MDA5 molecules (34). These oligomeric RLR CARD assemblies have been proposed to nucleate the formation of MAVS polymers (Figure 2D) (20, 25). Notably, the polymeric form of MAVS, but not its monomeric form, activates downstream RLR signaling (25). Moreover, once MAVS polymers have been nucleated they are self-propagating, drawing soluble-form MAVS monomers into the polymer. The MAVS CARD, even when isolated from the C-terminal and transmembrane domains, recapitulates this behavior *in vitro* (25). MAVS CARD polymers were recently found to consist of helical filaments (36), similar to those formed by the death domains of MyD88 (4, 46). The switch from a soluble form to a self-propagating helical fiber is reminiscent of amyloids and prions, and indeed MAVS CARD functions like a *bona fide* prion in yeast (47). Thus, MAVS has a prion-like mechanism of signal activation and amplification. ASC, the adaptor of the NLRP3 inflammasome, was recently shown to have a similar prion-like mechanism of signal transduction (47).

A transmembrane domain tethers MAVS to the mitochondrial or peroxisomal membrane. MAVS polymerization may therefore cause some remodeling of the membrane in these organelles (Figure 2D) (36). In support of this notion, MAVS facilitates cell death by disrupting the mitochondrial membrane potential and by activating caspases (48). Notably, the signaling output from MAVS is different depending on whether it occurs at the peroxisomal or mitochondrial membrane. Peroxisomal MAVS induces the rapid interferon-independent expression of defense factors, which precedes the activation of the principal interferon-dependent pathway by mitochondrial MAVS that amplifies and stabilizes the antiviral response (49). Thus, MAVS signaling is dependent on cellular localization, and peroxisomes are an important site of antiviral signal transduction (49).

## Regulation of RLR Signaling

The inflammatory response resulting from RLR signaling unavoidably occurs at a cost to normal tissue function. Multiple regulatory mechanisms have evolved to allow rapid activation, amplification, and inactivation of RLR signaling, and to achieve the optimal trade-off between the cost and benefit of the inflammatory response (50). Polyubiquitination has been one of the most extensively studied modifications of RIG-I and MDA5, so it is not surprising that E3 ligases and deubiquitinases have been implicated in modulating the RLR response. TRIM25, the most exhaustively studied E3 ligase, covalently attaches K63-linked polyubiquitin to RIG-I CARDs to initiate or promote signaling (26, 43). The E3 ligase Riplet has recently been identified as a necessary component of RIG-I signaling (51). USP21 negatively regulates RIG-I signaling by deubiquitinating RIG-I (52).

In addition to ubiquitination, phosphorylation is slowly emerging as an important regulatory mechanism for RLR signaling. Phosphorylation of Ser8 and Thr170 in the CARDs of RIG-I antagonizes RIG-I signaling (53, 54). Based on the crystal structure of RIG-I in complex with K63-linked diubiquitin (35), we expect phosphorylation of Ser8 but not Thr170 to interfere with ubiquitin binding. Phosphorylation of RIG-I CARD has also been proposed to inhibit recruitment of TRIM25 and MAVS (53, 54). The RIG-I phosphorylation sites are not conserved in MDA5, but MDA5 does have a suppressing phosphorylation site in its first CARD, at Ser88 (55). Conventional protein kinases Cα and β (PKCα/β) have been identified to be responsible for RIG-I phosphorylation (56). RIG-I and MDA5 are thought to be constitutively phosphorylated until presentation of viral RNA, at which time the RLRs must be dephosphorylated by phosphoprotein phosphatase 1 α and γ (PP1α/γ) (55).

Besides post-translational modification of the RLRs, RLR signaling is also modulated by several different proteins, derived both from the host and from pathogens. One such protein is the third RLR, LGP2. Because it lacks CARDs, LGP2 cannot activate MAVS; however, its ability to recognize dsRNA allows it to modulate the signaling capacities of RIG-I and MDA5. LGP2 downregulates signaling by RIG-I (32, 33). This activity was attributed to LGP2 competitively recognizing the same viral ligand as RIG-I. In contrast, LGP2 enhances MDA5 signaling (30, 33, 42). The molecular mechanism of this enhancement remains unclear, but LGP2 appears to facilitate recognition of viral RNA by MDA5 through interactions between the LGP2 CTD and RNA (41). Indeed, a recent study identified a specific picornaviral RNA ligand (in the antisense L region) to which LGP2 binds tightly, thereby stimulating MDA5 signaling (31).

The seemingly contradictory roles of LGP2 in RLR signaling remain an open question. The experimental approaches used to study LGP2 in relation to MDA5 and RIG-I have been different, potentially explaining some of the differences. As evidence accumulates for the opposing roles of LGP2 on RLR signaling, however, the emerging perspective is that LGP2 can control the balance between RIG-I and MDA5 responses during viral infection.

Pathogen evasion tactics against RLR-mediated immune response are extensive and occur at every level of signaling [reviewed in Ref. (57)]. A complete description of these tactics is beyond the scope of this review, so we highlight below a few representative examples of different modes of RLR evasion. MAVS is the primary target of viral factors for inhibiting RLR signaling. MAVS is cleaved by hepatitis C virus NS3/4A protease (28, 29), enterovirus 71 protease 2Apro (58), GB virus B NS3/4A (59), and coxsackie virus B 3C protease, which also cleaves TRIF (60). In a distinct mechanism of RLR signal inhibition, paramyxovirus V proteins disrupt the fold of MDA5 (61). Another major mechanism for evasion of the RLR innate immune response is masking or hiding of viral RNA ligands by viral proteins, such as VP35 from Ebola and Marburg viruses, which coat the ends and backbone of dsRNA to prevent RLR recognition (62–64). Similarly, nucleoproteins from arenaviruses bind to the ends of viral dsRNA and digest the RNA in a 3′–5′ direction, thereby making the RNA a weaker ligand for RLRs (65–68). Interestingly, MAVS was recently also shown to be under cellular control. A truncated variant of MAVS resulting from alternative translation initiation interferes with interferon production induced by full-length MAVS (69).

## Conclusion

RIG-I and MDA5 are the principal sensors of viral dsRNA in the cytoplasm. The interferon-dependent inflammatory response triggered by RLR ligand binding is one of the first and most important lines of defense against infection. RIG-I and MDA5 recognize distinct and complementary sets of viral dsRNA ligands. The molecular signaling mechanisms of RIG-I and MDA5 differ in some respects but also share certain key features. Differences include the sequestration of CARDs by RIG-I but not by MDA5 in the absence of ligand, the much greater propensity of MDA5 to form filaments along dsRNA, and the different contribution of K63-linked ubiquitin chains, which remains poorly defined for MDA5. Common features in RLR signaling include proximity-induced assembly of CARD oligomers, which serve as platforms to nucleate MAVS CARD polymerization, and signal amplification through the amyloid-like properties of the MAVS CARD. Together, the recent advances reviewed here shift the paradigm of RLR signaling away from the prototypical linear signaling cascade to a model in which signaling is activated by the cooperative assembly of an oligomeric signaling platform. The signal output depends on the cellular localization of MAVS (mitochondria or perixosome), and signaling is finely regulated by a multitude of cellular and pathogen-derived factors. Key outstanding questions include when, where, and how ubiquitin chains potentiate RIG-I and MDA5 signaling, exactly how RLRs interact with MAVS, and how LGP2 and other factors modulate RLR signaling.

### Outstanding questions

- Do K63-linked ubiquitin chains always participate in RLR signaling, or are they only required under specific physiological conditions that do not favor RLR filament formation?
  ○ Is the mechanism of action of K63-linked ubiquitin chains the same for RIG-I and MDA5?
- What are the molecular and structural bases of MAVS activation by RLR oligomers?
  ○ How do RIG-I CARD tetramers, stabilized by K63-linked ubiquitin, nucleate MAVS filament assembly?
  ○ How do MDA5 CARDs nucleate MAVS filament assembly? Does this process require K63-linked ubiquitin chains?
- What are the underlying molecular mechanisms for the opposite activities of LGP2 on RIG-I and MDA5 signaling?

## Conflict of Interest Statement

The authors declare that the research was conducted in the absence of any commercial or financial relationships that could be construed as a potential conflict of interest.
